# From facial expressions to bodily gestures: Passions, photography and movement in French 19^th^-century sciences

**DOI:** 10.1177/0952695115618592

**Published:** 2016-02

**Authors:** Beatriz Pichel

**Affiliations:** De Montfort University, UK

**Keywords:** chronophotography, Charles Darwin, Guillaume Benjamin Duchenne de Boulogne, history of emotions, photographic history

## Abstract

This article aims to determine to what extent photographic practices in psychology, psychiatry and physiology contributed to the definition of the external bodily signs of passions and emotions in the second half of the 19^th^ century in France. Bridging the gap between recent research in the history of emotions and photographic history, the following analyses focus on the photographic production of scientists and photographers who made significant contributions to the study of expressions and gestures, namely Duchenne de Boulogne, Charles Darwin, Paul Richer and Albert Londe. This article argues that photography became a key technology in their works due to the adequateness of the exposure time of different cameras to the duration of the bodily manifestations to be recorded, and that these uses constituted facial expressions and bodily gestures as particular objects for the scientific study.

## Introduction

In 1862, the physician Duchenne de Boulogne illustrated his *Mécanisme de la physionomie humaine* with photographs that reproduced the electrical experiments he had carried out on the faces of different patients. The aim of these localized faradizations was to determine the specific muscles involved in the expression of the passions. Some years later, around 1895, the photographer Albert Londe produced the chronophotographic series ‘Escape with terror’ as part of Paul Richer’s research on the physiology of movement. By showing a naked man running in profile, this series of 6 photographs attempted to make visible the external changes that passions provoked in the moving muscles.

Both projects utilized photography to study the influence of passions over muscles, but the resulting images were completely different. While Duchenne’s photographs focused on the face of the subjects, Londe’s series displayed the whole body (see Figure 2 and Figure 7, below). The photographic technologies employed in each case were also different. Duchenne operated with an 1850s camera and Londe had built a chronophotographic device equipped with 12 lenses that provided a succession of images in a short period of time.

Why were these two sets of photographs so different? Did these changes correlate with different conceptions of the passions and their expression? This article will answer these questions by mapping diverse approaches to the passions and the emotions that were developed in the related fields of psychology, psychiatry and physiology in France in the second half of the 19^th^ century. Studies have already demonstrated the mutual influence of physiology and psychology over modernism and the arts around 1900 (Silverman, 1989; Micale, 2004; Brain, 2008). However, the specific role of photographic practices in these cultural exchanges, particularly in relation to scientific approaches to the passions and the emotions, has been overlooked. This article will examine the production of several prominent contributors: Duchenne de Boulogne, Charles Darwin, L. Loreau, Paul Richer, Albert Londe and Georges Demenÿ. While Londe was a photographer, the rest were scientists working in different but related fields (medicine, natural history, neurology, psychiatry, physiology and psychology). This variety of approaches to the passions and the emotions accounts for the interdisciplinary perspective of this research, at the crossroad of the history of emotions, the history of medicine and photographic history.

The first part of this article will establish the basis for a dialogue between history of emotions and photographic history, while the following sections will demonstrate that this intersection is feasible and desirable by analysing 5 case studies. These cases trace the origins and challenges of the idea according to which emotions are mainly expressed on the face, and they identify with the instant capture by photography.

## History of emotions and photographic history

Thy Phu and Lynda Steer have remarked in ‘Affecting Photographs’, a special issue of *Photography & Culture*, that the photographic works of Duchenne, Darwin and Charcot have been extensively analysed. They argue, however, that ‘photography’s relationship to affect is more complex than this historically scientific focus suggests: besides describing emotions, photography also produces them’ (Phu and Steer, 2009: 237). These authors, alongside the contributors to the compilation *Feeling Photography* (Brown and Phu, 2014), have defended an ‘affective turn’ (Massumi, 2002), interrogating how affective reactions contribute to create photographic meaning.

This article will show that besides affecting the spectator, photographs have produced emotions as a particular kind of scientific object. In order to develop this idea, it will mainly draw on two traditions that have lately focused their analysis on scientific practices: photographic history (Cartwright, 1995; Tucker, 2005; Wilder, 2009) and the history of emotions (Bound Alberti, 2006; Dror, 1999a, 1999b, 2011; White, 2009a, 2009b). Both approaches share more than has been hitherto acknowledged and their intersection will prove very helpful for the historical inquiry.

The history of emotions is a multidisciplinary field of research that examines the shifting understanding and categories of the affective life, the changing lived experiences and the historical agency of emotions (Bourke, 2005; Frevert, 2011; Rosenwein, 2006). Regarding the first issue, Thomas Dixon (2003) has identified a turning point in the late 19^th^ century, when psychologists started to use the category ‘emotions’ to encompass all affective states. However, as Boquet and Nagy have argued, the French word ‘*émotions*’ does not correspond to the English ‘emotions’, as it generally refers to shocks of a very short duration, while ‘*passions*’ indicates more prolonged states (2011: 11–12). Moreover, French psychologists such as Théodule Ribot rejected the English reduction of the affective life to the ‘emotions’ (1906). Therefore, this article reserves the term ‘emotions’ to the history of emotions as a discipline, and to the cases in which authors such as Darwin, François-Franck and Dumas specifically used it.

The medical and scientific domains are receiving growing attention in this field. Not only have emotions been part of the scientific activity, as Paul White has argued (2009a), medical, psychological and physiological theories and practices in the laboratory have also created different and often overlapping paradigms within which emotional states have been historically understood. Particularly relevant for this article are the studies focused on the close of the 19^th^ century, when the application of technologies of inscription such as the cardiograph ‘generated, purified, quantified, measured, manipulated and…recorded emotions in visual and numeric form’ (Dror, 1999b). The tracings and outputs manufactured by these technologies cannot be considered as mere quantificational translations of emotions inasmuch as they materialized an entirely new paradigm.

Most of the physiologists working on emotions in this period embraced this approach, but the body did not only become an object of interest because of its internal operations. As it will be shown later, French physiologists such as Paul Richer also conducted research on the external modifications of the body in movement (Callen, 2003). Likewise, facial expressions became an object of scientific investigation in an ongoing dialogue if not direct confrontation with previous traditions such as physiognomy (Delaporte, 2010; Dupouy, 2011). Following this path of inquiry, this article contributes to the history of expressions (Jones, 2014) and gestures (Braddick, 2009) by analysing photographic practices set out by physicians, scientists and photographers at the turn of the century. With this objective, it follows the innovative approach of recent photographic history (Edwards, 2012; Edwards and Hart, 2004; Tucker, 2009). Unlike the canonical history of photography, concerned either about images (Sontag, 2003) or the political, social and economic structures that produce photographic meaning (Tagg, 1988), these works bring photography to the centre of historical analysis. They focus on the historical effects of material photographic practices such as taking, developing, exchanging, displaying, discussing, hiding, forgetting and looking at photographs.

This perspective allows a better understanding of the uses of photography in the sciences. Kelley Wilder has defended that ‘visualizing with photography…is never *merely* a matter of making visible the previously invisible’ (2011). In her analysis of Henri Becquerel’s photographs of radioactivity, Wilder shows that, by giving the rays a visual presence through the material reactions of the glass plates and the emulsions, the photographs also gave ‘radioactivity a materiality where it had not’ (ibid.). This and other works (Tucker, 2005; Brusius, Dean and Ramalingam, 2013) demonstrate the need to think about scientific photography as an instrument of observation and experimentation: as a tool for making knowledge and producing scientific objects rather than to merely illustrate them.

There is therefore a common ground between the history of emotions and photographic history that should be further developed. The focus on practices in photographic history involves the analysis of technologies, performances and images. It generates a complex space within which the shifting historical meanings of emotions can be understood in all their intricacy. This is particularly true for works in both fields exploring the idea that scientific objects and concepts are inseparable from theories but also from instruments, technologies and the practices applied to interpret the results. In this sense, this article will examine how photographic instruments gave a visual and material presence to particular understandings of emotions and passions by means of defining their bodily expressions. It claims that the photographic intervention invested expressions and gestures with qualities that were the result of the specificities of the devices that captured them –particularly their exposure time and sensibility of the plate’s emulsion.

## Facial expressions in Duchenne de Boulogne and Charles Darwin

The first two works that introduced photography as a tool for the scientific study of expressions were the aforementioned *Mécanisme de la physionomie humaine, ou analyse électro-physiologique de l’expression des passions* by the French physician Guillaume Benjamin Duchenne (de Boulogne), published in 1862, and *The Expression of the Emotions in Man and Animals*, by the British naturalist Charles Darwin, which appeared 10 years later. Both works have been extensively studied (Delaporte, 2003; Mathon, 1999a; Cuthbertson, 1990; Prodger, 2009; Donald and Munro, 2009; Watt-Smith, 2014: 39–81). Therefore this part will mainly focus on one particular aspect of their photographic projects: the time synchronization between the photographic camera and the expressions to portray.

Duchenne de Boulogne’s study belonged to a discourse on the representation of the passions in physiognomy that had been revived during the 19^th^ century (Hartley, 2001). He started his book reviewing the works of Camper, Lavater, Bell and Sarlandière, who had already used visual means to convey their theories on the expression of the passions through the facial muscles. Against them, Duchenne argued that expressions were produced by the simultaneous contraction of several muscles. Therefore, the physiologist should focus the experiments on identifying their movement. With this aim, Duchenne proposed the application of localized electricity to facial muscles and its reproduction through photography.

**Figure 1.**
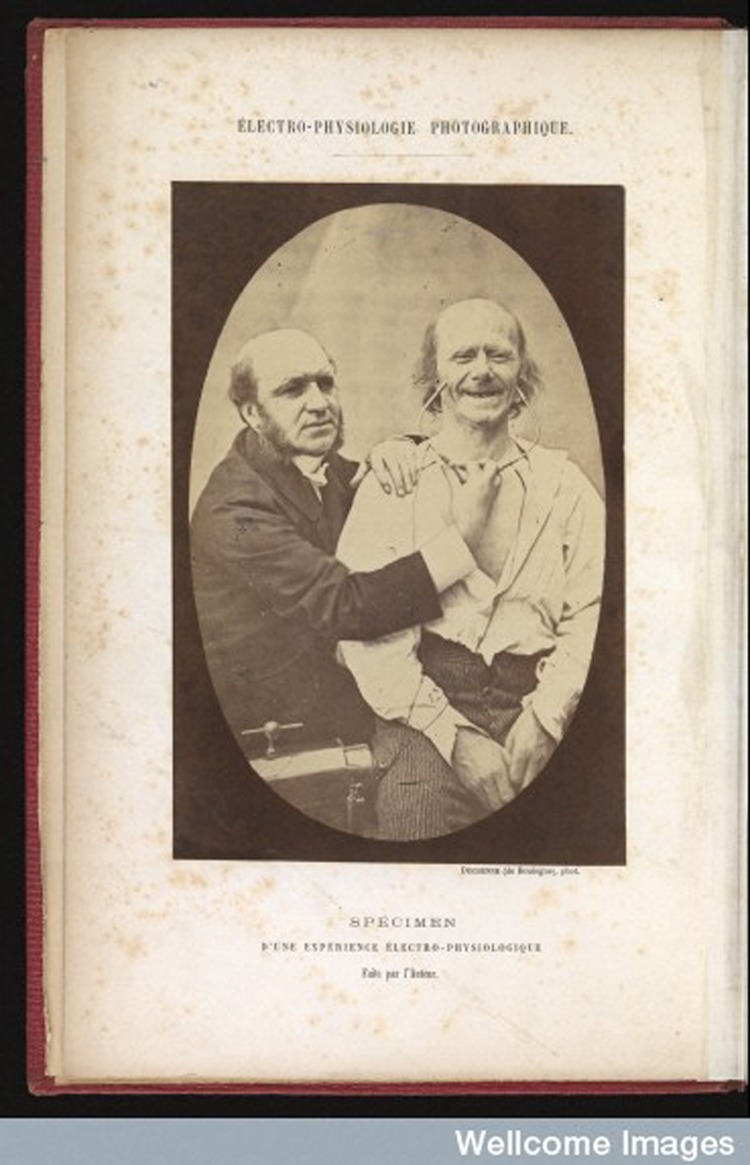
Duchenne de Boulogne, ‘Electro-physiologie photographique’, from his *Mécanisme de la physionomie humaine* (1862). Wellcome Images.

**Figure 2.**
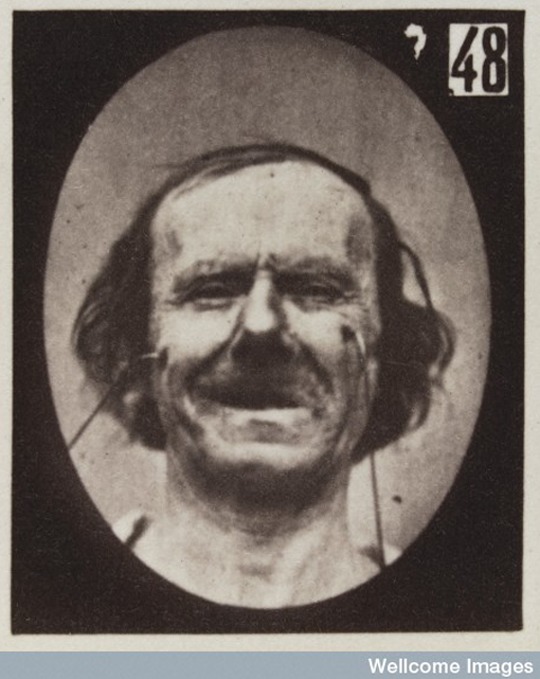
Duchenne de Boulogne, Figure 48, from his *Mécanisme de la physionomie humaine* (1862). Wellcome Images.

The photographs that illustrated *Mécanisme* had been taken around 1856, as part of the research he presented for the French national Volta Prix in 1856 and 1857 (Duchenne, 1857/ Delaporte, 2010). The original plates displayed the face of the subject of the experiments, the upper half of his body and sometimes Duchenne with his assistant manipulating the electrodes, as can be seen in Figure 1. This kind of portrait composition survived only in the first photograph of *Mécanisme*, in which Duchenne posed next to an old man and the faradizing device. The rest of the photographs were cropped in order to direct the viewer’s attention to the facial changes, as shown in Figure 2. Some editions of *Mécanisme* even removed the bodies of the model and the physician from the photographs belonging to the aesthetic part, in which bodily gestures complemented the narrative conveyed by the facial expressions. The cropped figures were also arranged in synoptic tables that reproduced the same expression up to three times, masking different parts of the face. Duchenne was equally involved in the production of the photographs. Whether it was he or Adrien Tournachon who operated the camera, Duchenne ‘directs, conceives the project, selects the intensity and duration of the electrical courant, settles the subject, directs his gaze and the position of the mouth, and decides the moment’ to take the photograph (Mathon, 1999b: 15). A key element in this process was the artistic use of light, which highlighted the expressive lines of the model – a great achievement taking into account that he relied on natural light.

Duchenne’s engagement in this process reveals that, together with the faradization machine, photography became an essential tool in the definition of facial expressions. The centrality of photography in the project is manifest in Duchenne’s memoir for the Volta Prix, where he stated the rationale for sending a photographic album instead of engravings or illustrations as his predecessors had traditionally done. These explanations seemed necessary in a period when photography had just started to be used in science, and very few scientific books had been illustrated with photographs in France (Sicard, 1995). Duchenne argued that his experiments were of a visual nature, as he had repeated these experiences in front of hundreds of people, and they had always convinced them all. Therefore he needed images to disseminate their results among people who were not able to witness them. Skilled artists had first tried to copy the expressions produced by the faradizations, but they had not been able to do so because these expressions lasted only for a few seconds. As a consequence of their failure, Duchenne argued that only photography, ‘as truthful as a mirror’, could attain ‘such desirable perfection’ (1857: 58).

Two kinds of arguments supported the choice of photography. First, by qualifying photography as ‘truthful’, Duchenne was alluding to the visual properties of photographs as epistemological objects. In this regard, he insisted, ‘the vision of the photographic figures that represent, in a truth-to-nature way, the expressive traits that are specific to the muscles of the passions could teach much more than long considerations and descriptions’ (1857: 58). For Duchenne, therefore, the mechanical procedure of the camera guaranteed that the image would mirror what had happened in front of it. However, his position cannot be directly identified with the ideal of mechanical objectivity described by Daston and Galison (2007). While the veracity of the photographs was based on the mechanical functioning of the device, the aforementioned manipulations of the photographs reveal that the scientist could in fact intervene in the process in order to make the image as true-to-nature as possible.

Second, the comparison between artists and photographers suggests that the difference between them did not lie in the images they produced, but in the technology they used. In particular, Duchenne stressed the relationship between the duration of the event to be recorded and the speed of the instrument that captured it. Duchenne’s faradizations lasted only for a few seconds, but unlike artists, the camera also needed a few seconds to capture the object that was in front of it. Therefore, the photographic camera was a suitable instrument not only for the truthful images it produced, but also because its exposure time coincided with the seconds during which the electrodes could hold the expression. This time was enough to record the results of faradization but not other gestures, as the images of blurred heads of Duchenne and his assistant suggested.

This synchronization, even if not exact, between the camera and the electrical device became the key element in the photographic production of facial expressions. The electrically stimulated expressions could look natural, but they were expressions held in time. Even when the expression had not been produced by faradization, like in the case of the true smile (Duchenne, 1862: 56), it had been paralysed by the photographic image. As obvious as this fact is, it reveals the inherent paradox of Duchenne’s project and one of the main characteristics that pervaded later understandings of expressions. While movement was the condition for the study of expressions, it was removed from the final evidence of that study. In spite of the title, Duchenne did not reproduce the mechanism of the facial expressions. He was not interested in showing the process of producing the expressions (the muscles contracting), but in the instant during which the expression was held. As a result, it was precisely this particular paralysed instant produced by faradization and photography together that became known in the scientific tradition as the ‘expression’.

Despite the novelty of his work, Duchenne remained relatively unknown. These photographs became famous only with their reproduction in *The Expression of the Emotions in Man and Animals*, by Charles Darwin, 10 years later. The British naturalist had started to pay attention to emotional expressions when his first son was born in the 1830s, but it was not until the 1870s that these observations became part of his published research. What was supposed to be a chapter of *The Descent of Man* (1871) became a book of its own due to the extensive material that he had collected. Besides the comments in his notebooks, Darwin had gathered an important number of visual examples and had sent questionnaires to collaborators and other researchers around the world (Prodger, 2009; White, 2009b).

The origin of Darwin’s photographic collection was varied (Prodger, 1998). Besides the famous collaboration with the Victorian artistic photographer Oscar Rejlander, Darwin also owned photographs sent by the psychiatrist James Crichton-Browne and the photographer Giacomo Brogi (collaborator of the Italian physiologist Paolo Mantegazza), as well as portraits from different English photographic studios.

This diversity demonstrates one of the main differences from Duchenne’s work. Darwin collected photographs inasmuch as they were valuable observations of human and animal expressions. Therefore, the photographs lacked a standard protocol of production. Even in the cases when Darwin had asked Rejlander to photograph particular expressions whose representation was especially difficult to find, he did not give any instructions on the ways in which the pictures should be taken (Prodger, 2009). Unlike Duchenne, who personally produced all the photographs in the same conditions and cropped the images to reinforce the continuity among them, Darwin’s collection was an amalgam of faces and bodies.

The reasons why Darwin had turned to photography were quite similar to those argued by Duchenne. As he recognized, ‘the study of expression is difficult, owing to the movements being often extremely slight, and of a fleeting nature’ (1872: 13). Then, again, the question was to find an adequate technology that could synchronize with these slight, fleeting movements. According to Darwin, ‘it is easy to observe infants whilst screaming; but I have found photographs made by the instantaneous process the best means for observation, as allowing more deliberation’ (ibid.: 148). Once again, photography became a valuable tool not solely for the images it provided, but also because it fitted the time that was needed to capture the expression. The advances on wet collodion plates had allowed the reduction of exposure times to the instant; but photography still had the same function: to freeze movement in a single shot. Like Duchenne, Darwin explained in the text how the muscles contracted, but the photographs depicted only the instant that defined and identified the expressions, leaving out of representation the movements that led to that instant. This reinforced the sense that the ‘expression’ was what had been captured by photography by freezing time. But it also introduced another idea: the concept of the ‘natural’ expression. The problem now was not just capturing ‘fleeting states’, but also seizing real expressions.

The emphasis on naturalness demarcates another key difference with Duchenne, for whom photography was an experimental tool. The search for accurate representations of natural expressions led Darwin to draw on multiple sources, from engravings to paintings and commercial photographs: Duchenne’s photographs were in fact reproduced in the book as woodcuts in which the electrodes had disappeared. Stressing the naturalness of expressions has been interpreted as a strategy to connect with the Victorian reader, moderating the impact of the evolutionary links between human and animal expressions (White, 2009b; Voss, 2010). Naturalness was, therefore, a concession to Victorian sentimental values (Dupouy, 2011), but also the key to his photographic project and one of its most long-lasting effects.

**Figure 3.**
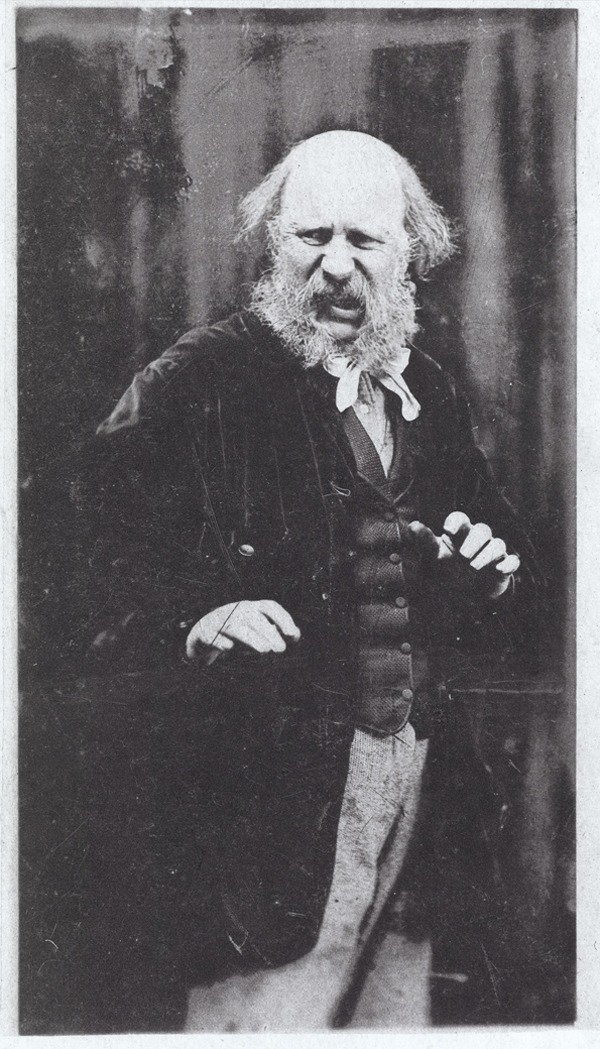
‘Rejlander – Expression of Disgust’, from Charles Darwin, *The Expression of the Emotions in Man and Animals* (London: John Murray, 1872), p. 255. Wellcome Images.

During the 1870s it became common in the photographic press to discuss the best methods to capture real expressions in portraiture. The *British Journal of Photography* published articles interrogating whether the very act of taking a portrait could affect the sitter’s expression. These texts explained that the act of taking a picture was usually annoying, sometimes even painful, and made people feel nervous (Sheehan, 2011). Most of these commentators advised photographers to interact with the sitters to make them forget that they were at the photographic studio, while others introduced changes in the camera, so the movement and noise of the shutter did not distract them (see *British Journal of Photography*, 1872a, 1872b). These strategies were intended to minimize the intervention of the camera, particularly in the case of children –the most difficult subjects besides animals. Rejlander was aware of these problems, and also tried to connect with the sitters, using even his wife as a model. He also posed to recreate expressions of astonishment, fear, disgust, indignation, indifference and surprise (Figure 3). As he recognized, ‘It is very difficult to get, at will – those expressions you wish – Few have the command of imagination to appear real – In time, I might catch some – So I have tried in propria persona’ (Darwin, 2012[1871]). Naturalness referred therefore to expressions that allegedly reflected the emotion felt by the subject. However, photographs like Figure 3 demonstrate that even ‘natural’ expressions could be performed at will by the subject in front of the camera.

While Duchenne defended the universal language of the passions created by God, Darwin understood expressions as an evolutionary legacy or reflex actions. In consonance, they also used different photographic technologies. Photography became an integral part of Duchenne’s experiments, whereas it served to collect impressions of natural expressions in Darwin’s project. Despite these differences, both turned to photography because it was the medium that fitted best the time needed to capture the expression. As a result, they consolidated two ideas: that emotions and passions were mainly expressed on the face, and that the particular instant captured by photography defined and identified how the expressions appeared. The following sections will show how later developments in France precisely challenged these two postulates.

## The passionate gestures: The iconography of the Salpêtrière

Darwin’s theory of the expression of emotions was soon appropriated by practitioners of the new discipline of experimental psychology in Britain, France and America. In France, this school was represented by the work of Théodule Ribot, the first to teach courses on experimental psychology at the Sorbonne (1885–8) and later at the Collège de France (1888–1901) (Nicolas and Charvillat, 2001). Drawing on Darwin and other psychologists such as William James, Ribot argued that passions, emotions and affective states could be understood as bodily movements that were manifested either externally by means of expressions, gestures and attitudes, or internally, through secretions, the breath, or the pulse (Ribot, 1906). This new approach distanced itself from previous conceptions of the passions and theories on the intellectual origin of emotions (Dixon, 2003).

These theoretical changes paralleled the introduction of new methods of research. Instruments such as the graphic method, which allowed the transcription of bodily changes associated with the emotions in a quantifiable way, were introduced in the laboratory by experimental psychologists such as Alfred Binet (Dror, 2011). Photography was mostly abandoned as a scientific tool in this field, but previous theories grounded on photographic findings were not discarded.

Photography, however, did not disappear from the scientific study of expressions. A few years after the publication of *The Expression*, photographs illustrated the widely known *Iconographie photographique de la Salpêtrière*. Under the direction of the neurologist Jean-Martin Charcot, the physician Désiré Bourneville and the intern Paul Regnard edited a journal to disseminate the portraits of hysterical women taken at the hospital. The *Iconographie* published 4 numbers from 1875 to 1880, when Bourneville left. It reappeared as the *Nouvelle iconographie de la Salpêtrière* in 1888 with Albert Londe as the director of the photographic service.

Much has been written about the spectacular and even theatrical character of Charcot’s lessons (Gordon, 2004; Justice-Mallow, 1995), as well as on the photographs that documented the hysterical attacks of female patients (Baer, 2002; Didi-Huberman, 1982). Unlike these works, this section will concentrate on the ways in which the photographs produced at the Salpêtrière introduced a key question in the scientific debates on emotions: the role of bodily gestures in the performance of the passions.

Gestures became an object of study primarily during the third phase of the hysteric-epileptic attack, the so-called ‘*attitudes passionnelles*’ [passionate attitudes] (Richer, 1885: 89–116). More interesting for the purpose of this article are the photographs that documented the three series of experiments on the neuromuscular hyperexcitability of hysterical subjects under hypnosis carried out by Charcot and Richer between 1881 and 1885. These experiments led to the revival of hypnosis as a scientific method of research. A supporter of pathological medicine, Charcot believed that pathological states such as hysteria could reveal information on the normal functioning of the body (Carroy, 1991). These experiments would particularly ‘contribute to solve some of the higher problems in physiology and even in psychology’, as they would reveal the mechanisms behind the automatic responses of the nervous system (Charcot and Richer, 1881a: 32).

**Figure 4.**
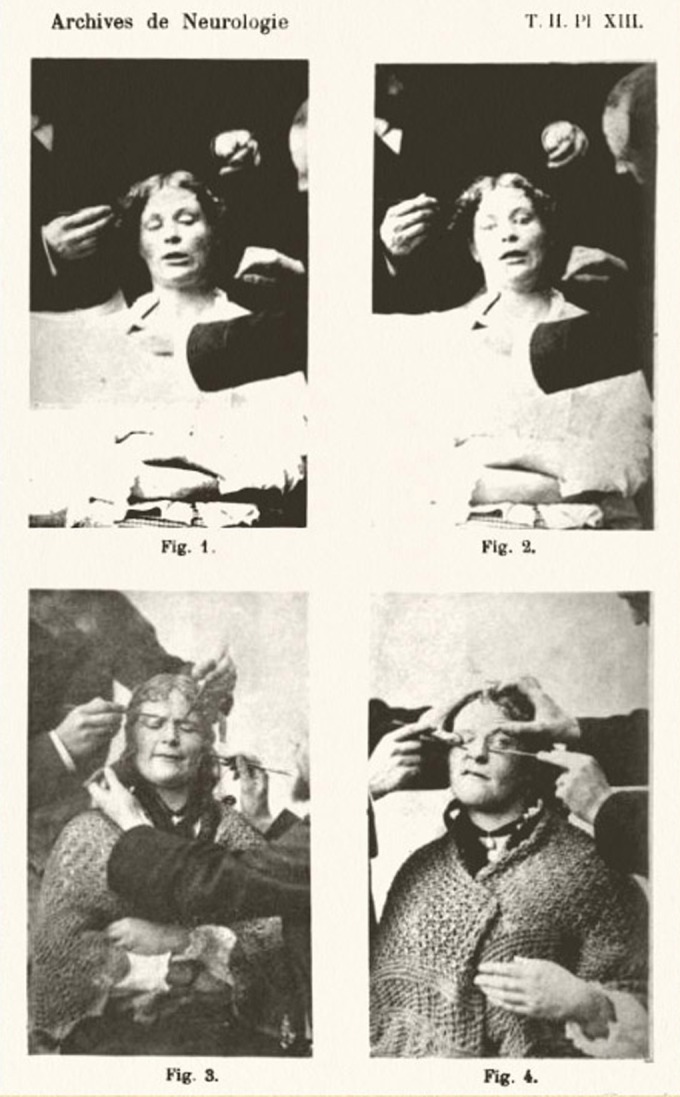
L. Loreau, plate XIII, vol. II, *Archives de neurologie* (1881). Jubilothèque, UPMC.

The first series of experiments was published between 1881 and 1882. Charcot and Richer (1881a, 1881b, 1882) reinterpreted Duchenne’s work by pressing the muscles of the face of different female patients with a small piece of wood. The facial contractions achieved through this pressure demonstrated that, in a state of hypnosis, the excitability of the muscles increased so much that localized faradizations were unnecessary to stimulate them. Therefore, these experiments lacked the electrical component of Duchenne’s project, but shared with it a common interest in the anatomical origin of expressions, as well as the use of photography to document it. Loreau, modeller at the museum of pathological anatomy at the Salpêtrière, was in charge of portraying the female patients looking at the camera while the doctors manipulated the batons over their faces (Figure 4). This formal composition recalls the photographs that illustrated Duchenne’s first works, examined in the previous section. Furthermore, Loreau’s photographs were also intended to serve as visual evidence of the results of the experiments, and were reproduced in the article.

**Figure 5.**
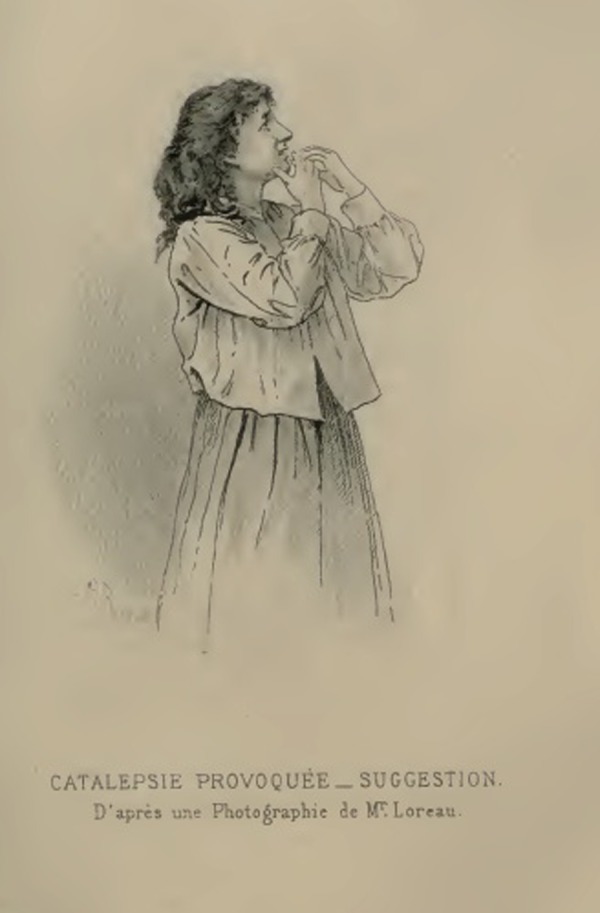
‘Catalepsie Provoquée – Suggestion’, d’après une Photographie de M. Loreau: from Paul Richer, *Études cliniques sur la grande hystérie* (1885). Not in copyright.

A second series tested how bodily gestures (the ‘attitude’) were linked to facial expressions. Its aim was to demonstrate that the expressive movements imposed on different body parts in subjects in a cataleptic trance led to secondary movements on the face that completed the expression. For example, Richer explained, ‘a tragic attitude impresses a tough appearance to the physiognomy, the eyebrow contracts. On the contrary, if we approach the hands to the mouth, like in the act of sending a kiss, a smile immediately appears’ (1885: 669). Once again, Loreau photographically documented the experiment, but the text was instead illustrated with two drawings made after the photographs (Figure 5). This transformation had become a common practice at the Salpêtrière thanks to the artistic skills of Richer, who joined the team precisely because of his ability to make accurate depictions of the phenomena under study (Ruiz-Gómez, 2013).

The second edition of Richer’s *Études cliniques* included a third series of experiments performed between November of 1881 and 1885, in which elements of the two previous experiments were combined. It consisted of the application of electrodes connected to a Dubois-Raymond electrical machine to the faces of cataleptic patients. These localized faradizations provoked facial expressions by contracting facial muscles other than the stimulated ones, as well as the attitude – the bodily gestures that completed the expression. These experiments were performed in two stages. While the first series of 1881 photographed the patients when the electrodes had been removed from their faces, in 1884 Richer decided to leave them. In this way, the images could show the exact point that had been faradized (1885: 671).

**Figure 6.**
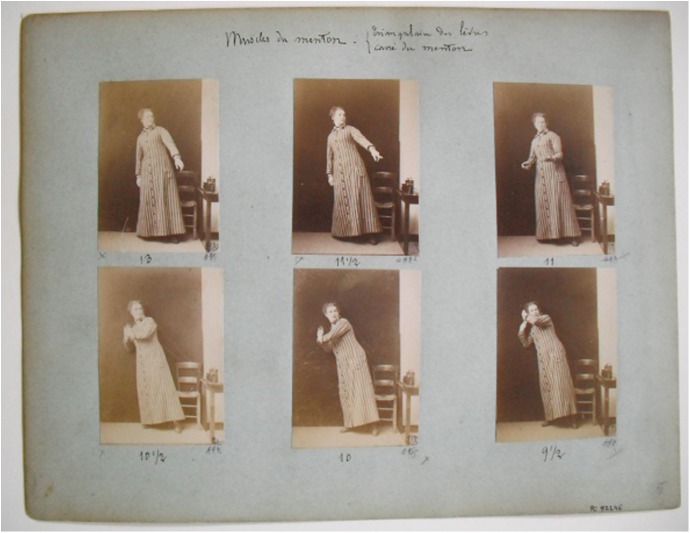
Ph 846. Faradization des muscles du menton. Albert Londe, *c*. 1885. École Nationale des Beaux Arts, Paris. Reproduced with permission of ENSBA, Paris.

According to Richer, the patients became ‘expressive statues’, as the pose achieved by faradization was held for some instants (1885: 670). However, Londe recognized in 1884 the difficulty of capturing these expressions, and particularly the nuances of intensity. Interestingly, Londe insisted that artists would have not been able to copy them because these poses only lasted for a very short period of time (Londe, 1884: 10). Once again, photography was presented as the most suitable technology for the reproduction of expressions because of the adequacy of its exposure time to the duration of the gestures, as well as the higher sensibility of the emulsions of the plate. In this case, Londe had used the stereoscopic camera he had built, which allowed him to take two images in the same plate in only one second, indoors and under bad weather conditions (Londe, 1884). These photographs were then mounted as single pictures on sheets that grouped the experiments according to the group of facial muscles that had been stimulated. The disposition of the portraits became a rhetorical device intended to show that applying different degrees of electricity led to correlative qualitative differences in the expression, as can be seen in Figure 6. Although *Études cliniques* included only the drawings made after the photographs taken in 1881, Londe’s treatise on medical photography published both the drawings and the original photographic series (Londe, 1893: 92–3).

This procedure and the experiment sound familiar; however, they introduced some key variations. According to Richer, they had gone even further than Duchenne. The movement of the limbs proved that faradizations acted on the surface of the muscles, but also that the centripetal nerves communicated the stimulation of facial muscles to the brain, and from there the movement was conducted to the secondary gestures (Richer, 1885: 671). Precisely because of this focus on internal bodily processes, documenting only the surface of the body was not enough. As Richer recognized, even if the truthfulness of the expressions was striking, it did not mean that the ‘intimate self’ was reacting and feeling the passions associated with the expressive performances. This is why, along with the drawings, Richer also included the inscriptions produced by a pneumograph and a cardiograph. They showed that the breath and the pulse of the patients barely changed when they were stimulated under hypnosis (ibid.: 680).

This explanation in neurological rather than anatomical terms reflected changes in the conception of the external manifestations of the passions. Against Duchenne and Darwin, the experiments performed in the context of the clinical study of hysteria revealed the radical importance of bodily gestures in the understanding of both the disease and the expression of the passions. Produced by the experiment, these gestures were closer to Duchenne’s figures than to Darwin’s natural expressions. Notwithstanding, they revealed the automatic, nervous reactions of the normal body –and ultimately, the somatic nature of the passions.

This defence of both the internal and the external bodily processes led furthermore to a reconceptualization of the role of photography in the studies on expression. On the one hand, photographs could no longer be the only visual evidence provided. They had to be completed by inscriptions produced by the graphic method that quantified the changes which occurred within the body. On the other hand, capturing the gestures posed a problem to photography as it had been practised thus far because it involved dealing with the question of movement and its visualization through a sequence.

Due to his practice as director of the photographic laboratory at the Salpêtrière, where he had to capture ‘trembling’ and ‘attacks’ (Londe, 1883: 127), Londe was well aware of this problem. According to him:

It is possible to capture the patient and to immobilize him by means of the instantaneous photography. However, these pictures taken randomly only represent one of the phases of the movement, a phase that our eye might not even perceive because of its rapidity. We will obtain a document, but we cannot accept that this document alone is able to show what we have perceived. (Londe, 1893: 96–7)

Unlike previous scientists and photographers, Londe insisted that the photograph of one particular instant could not define the entire gesture. Any selected moment would be a random choice and not the summary of all the movements that led into and resulted from it. For the first time in this context, photography did not seek to capture the instant, but the movement.

These theoretical principles materialized in the pictures Londe took after his arrival at the Salpêtrière in 1882. These photographs were intended to depict all the phases of the hysterical attack. With this goal Londe arranged them in a chronological order that emphasized the temporal continuity among the represented events (Bernard and Gunthert, 1993: 99–136). Photographs thus stopped being isolated items to become fragments of a series within which they made sense. These serial compositions of instantaneous pictures gave the impression of movement, but they were still random instants. According to Londe, these plates were not enough: ‘We need a special device that allows taking a certain number of pictures within particular intervals as close or apart from each other as we want them to be’ (Londe, 1883: 127). This special camera would ‘give successive images reproducing all the phases of movement’ (ibid.: 87).

## Capturing movement: Londe’s and Demenÿ’s chronophotography

Londe presented his first photo-electrical camera in 1883 before the Société Française de la Photographie. Inspired by the so-called ‘*fusil photographique*’ invented by the astronomer Jules Janssen in 1876, the 9 shutters of his device captured 9 images arranged in a ring on the same plate (Londe, 1883; Bernard and Gunthert, 1993: 123). Unlike other chronophotographic devices that worked with fixed times, this camera allowed the manual regulation both of the time intervals between each exposure and of the shutters’ speed, which made it particularly adequate to portray hysterical attacks (Londe, 1883: 127). Ten years later, Londe built a new chronophotographic camera. This second device provided images of a bigger format (8/8), which facilitated the projection and enlargement of the prints (Londe, 1893: 111). It had 12 shutters that were arranged in 3 parallel lines. The plate provided, therefore, 12 images organized in 3 rows of 4 images each. Once again, its mechanism allowed the combination of 4 different shutters’ speed with varied intervals between each pose (ibid.: 113–15).

Londe applied this device to Richer’s research on the physiology of movement. In line with studies on the relation between posture, health and evolution (Gilman, 2014), *Physiologie artistique de l’homme en mouvement* (1895) examined the mechanism of the muscles and the bones as well as the external modifications produced by physiological changes in muscles. The movements studied in this treatise included walking in normal and pathological ways, jumping, going upstairs and downstairs, and several sports exercises. The passions were not an object of analysis on their own, but Richer included the ‘expressive gait’. According to him, men’s goals interfered with their gait (Richer, 1895: 302). As he noted: ‘The gait will always reflect the passions of the man, and depending on whether he is lazy, enthusiastic, sad, happy, humble or proud, the gait will acquire particular signs’ (1901: 215). This was particularly important for artists, as they rarely represented men whose only purpose was walking.

The physiologist recognized that a complete study of the expressive gait would require a full investigation of the passions and their means of expression, a task that he would not undertake. Nevertheless, he sketched the analysis of the enthusiastic gait and the sly gait. For instance, the enthusiastic gait was that of ‘the ancient warrior coming back home after a victory’ or ‘the common man singing la *Marsellaise*’ (1895: 302). His description of the body stressed the particularity of the leg’s movement rather than the face as the most singular feature due to the exaggeration of the steps and the flexion of the leg when stepping onto the floor. Although these analyses were grounded on chronophotographs, the illustrations of *Physiologie artistique* were, as usual, drawings made by Richer after Londe’s photographs. Furthermore, he reproduced only one image that displayed the most remarkable features of the pose instead of the whole series.

**Figure 7.**
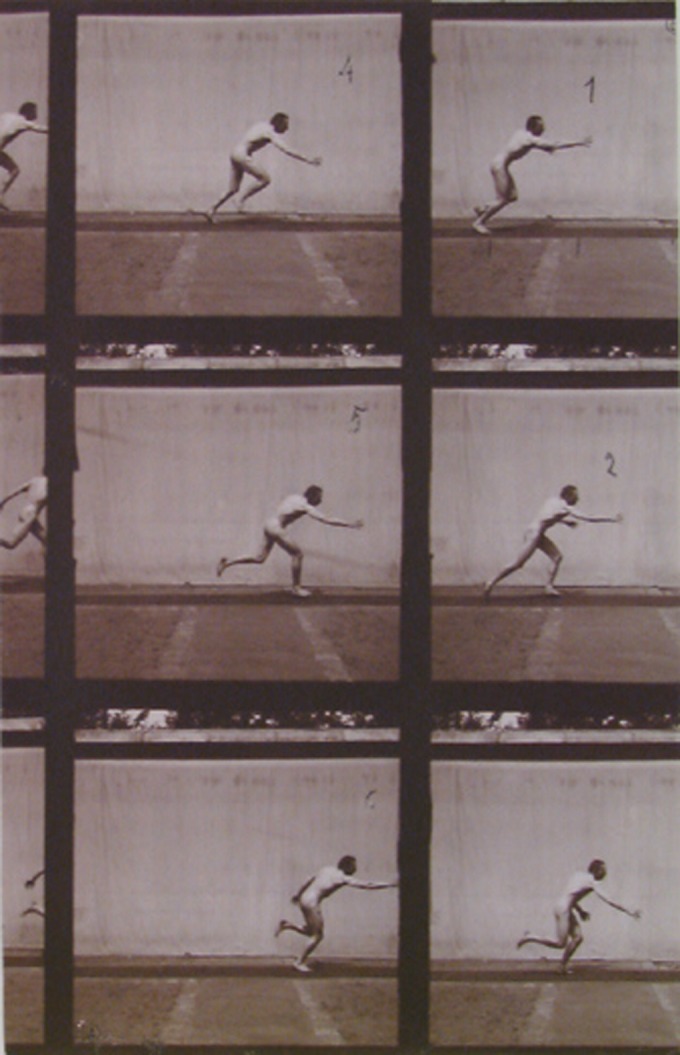
Ph462 Albert Londe, ‘Fuite avec effroi, vue latérale’. Reproduced with permission of ENSBA, Paris.

The chronophotograph ‘Escape with terror’ addressed at the beginning of this article (Figure 7) belonged to this series and was later fully reproduced in Richer’s *Nouvelle anatomie artistique* (1921: 25). The use of chronophotography as a research method provided not only images of the previously unseen, but also new tools for thinking about bodily movement and the nature of gestures. These series in particular examined normal passionate states, but the physiological explanation of the muscular reactions arrived at the same conclusions on the automatism of bodily responses that had been developed in the studies on hypnosis. Therefore, they reinforced the two main ideas developed earlier at the Salpêtrière: the expression of the passions through the movement of the whole body, and the need for photography to capture as many phases of the movement as possible. Precisely because of this, they challenged the very principles established by Duchenne and Darwin: the prominence of facial expressions and their definition by the instant captured by photography.

Londe and Richer were not the only ones working with photography in scientific studies on expressions at the turn of the 19^th^ century. In 1892, Georges Demenÿ, assistant of the renowned physiologist and chronophotographer Étienne-Jules Marey, experimented with chronophotography to record facial expressions. The two series of ‘living portraits’ that he made captured several stages of the movement of his lips while he was saying ‘*Je vous aime*’ and ‘*Vive la France*’ (Demenÿ, 1892a, 1892b). Although Demenÿ was not interested in the physiological study of the passions per se, he defended the benefits of the chronophotography of expressions for commercial and vernacular photography. As he explained in *La photographie de la parole*: ‘In normal conditions, the successful portrait is only possible for the calm expressions of the physiognomy. Good laughs and exalted passions become grimaces without any relation to reality when we try to represent them’ (1892a: 3).

Recalling Darwin’s concern, Demenÿ sought to capture real expressions. But, like Londe, he insisted that ‘if we accept only one instantaneous image, it usually happens that we capture an unstable attitude of the movement that the eye cannot naturally perceive’ (Demenÿ, 1892a: 3). Demenÿ took then a step further, arguing that this series of instantaneous images should not only analyse movement, but also synthetize it by means of the zootrope in a single moving image.

Therefore, at the beginning of the 20th century it was technically possible to make photographic studies both of facial expressions and of bodily gestures that acknowledged their movement without reducing them to one instant. However, chronophotography was not further used in this field. Neither Demenÿ nor Londe continued this line of their work. Even the physiologist Charles-Émile François-Franck, Marey’s student, did not follow his mentor. Most of the photographs he collected to illustrate the two courses he taught at the Collège de France on the expression of emotions (1901–2), came from Duchenne, Darwin and other French alienists who used instantaneous photography (Huetz, 2014). In the same line, the psychologist Georges Dumas illustrated his works on the physiology and the psychology of the smile with instantaneous photographic portraits (Dumas, 1905, 1906).

## Conclusions

The different case studies analysed in this article have been chosen to demonstrate that photographic images of expressions and gestures were produced by means of the intersection of particular theories on the origin and manifestation of passions and emotions on the one hand, and the use of specific photographic technologies on the other. Duchenne de Boulogne turned to photography because he needed to record the electrophysiological experiments that would reveal the language of the passions, while Darwin collected instantaneous photographs because he was interested in the natural expressions as they were captured in studio photography. Richer and Charcot needed photographic series that reproduced the pathological gestures produced in hysterical conditions, and Richer and Demenÿ used chronophotography to capture the expressions in movement. The resulting images cannot be therefore interpreted as mere representations. The photographic intervention constructed the materiality of expressions and gestures in relation to two sets of concepts: the face and the body, and the instant and the movement. The return to facial expressions at the beginning of the 20th century demonstrates how powerful the photographic rhetoric of the instant *would become*.

These case studies shared a common thread: photography was selected as the technology to capture expressions and gestures because of the temporal correspondence between the duration of the event to be reproduced and the time needed by the camera to capture it. This idea provides a new perspective on reasons why photography became such a crucial instrument in scientific studies at the turn of the 19^th^ century. Much has been written on the objective character of scientific photographs, but the ideal of mechanical objectivity was always contested, never fully achieved and, as the examined case studies demonstrate, sometimes secondary. This article has shown that the use of photography emerged for pragmatic reasons: the choice of photographic technologies depended on the particular needs of each study. This perspective, which considers photography as another instrument in the laboratory, the clinic, or the studio, precisely opens up a dialogue between the history of emotions and photographic history. It stresses the specificity of the photographic intervention while it acknowledges the historicity of the practices and understandings of the emotions.

## Note

This article was first presented as a keynote at the METABODY Forum conference ‘Histories and Ontologies of Gesture–Movement’, Universidad Autónoma de Madrid, 11 July 2014. I warmly thank Eva Botella and Jaime del Val for their kind invitation. I would also like to thank my colleagues at the PHRC, de Montfort University, for their incredible support during two years of stimulating conversations. Further thanks go to the three anonymous reviewers for their insightful comments.
